# Surgical trends and regional variation in Danish patients diagnosed with lumbar spinal stenosis between 2002 and 2018: a retrospective registry-based study of 83,783 patients

**DOI:** 10.1186/s12913-023-09638-7

**Published:** 2023-06-20

**Authors:** Rikke Krüger Jensen, Christian Volmar Skovsgaard, Dorthe Schøler Ziegler, Berit Schiøttz-Christensen, Rune Mygind Mieritz, Andreas K. Andresen, Jan Hartvigsen

**Affiliations:** 1 Center for Muscle and Joint Health, Department of Sports Science and Clinical Biomechanics, University of Southern Denmark, Odense, Denmark; 2 Chiropractic Knowledge Hub, Odense, Denmark; 3 DaCHE – Danish Centre for Health Economics, Department of Public Health, University of Southern Denmark, Odense, Denmark; 4 Medical Spinal Research Unit, Spine Centre of Southern Denmark, University Hospital of Southern Denmark, Middelfart, Denmark; 5 Research Unit of General Practice, Department of Public Health, University of Southern Denmark, Odense, Denmark; 6 Department of Neurosurgery, University Hospital Odense, Odense, Denmark; 7 Spine Surgery and Research, Spine Centre of Southern Denmark, Lillebaelt Hospital, Middelfart, Denmark

**Keywords:** Lumbar spinal stenosis, Surgery, Decompression, Fusion

## Abstract

**Background:**

Lumbar spinal stenosis (LSS) is the most common reason for spine surgery in older people. However, surgery rates vary widely both internationally and nationally. This study compared patient and sociodemographic characteristics, geographical location and comorbidity between surgically and non-surgically treated Danish patients diagnosed with LSS from 2002 to 2018 and described variations over time.

**Methods:**

Diagnostic ICD-10 codes identifying patients with LSS and surgical procedure codes for decompression with or without fusion were retrieved from the Danish National Patient Register. Patients ≥ 18 years who had been admitted to private or public hospitals in Denmark between 2002 and 2018 were included. Data on age, sex, income, retirement status, geographical region and comorbidity were extracted. A multivariable logistic regression model was used to calculate the relative risk for surgically versus non-surgically treated LSS patients using the total population and subsequently divided into three time periods. Variations over time were displayed graphically.

**Results:**

A total of 83,783 unique patients with an LSS diagnosis were identified, and of these, 38,362 (46%) underwent decompression surgery. Compared to those who did not receive surgery, the surgically treated patients were more likely to be aged 65–74 years, were less likely to have comorbidities, had higher income and were more likely to reside in the northern part of Denmark. Patients aged 65–74 years remained more likely to receive surgery over time, although the difference between age groups eventually diminished, as older patients (aged ≥ 75) were increasingly more likely to undergo surgery. Large variations and differences in the relative risk of surgery were observed within and between the geographical regions. The likelihood of receiving surgery varied up to threefold between regions.

**Conclusion:**

Danish patients with LSS who receive surgery differ in a number of respects from those not receiving surgery. Patients aged 65 to 74 years were more likely to receive surgery than other age groups, and LSS surgical patients were healthier, more often retired and had higher incomes than those not undergoing surgery. There were considerable variations in the relative risk of surgery between and within geographical regions.

**Supplementary Information:**

The online version contains supplementary material available at 10.1186/s12913-023-09638-7.

## Introduction

A diagnosis of lumbar spinal stenosis (LSS) is the most common reason for spine surgery in people aged 65 years and older [1]. Rates of spine surgery rates vary widely internationally and nationally and are among the most variable of all surgeries, with a difference between states in the US ranging from 30 to 132 per 100,000 [2–5].

In Denmark, the total number of surgeries for LSS varied substantially between 2002 and 2018 [6], probably because of a lack of consensus among surgeons regarding when surgery is appropriate [7]. More recent guidelines suggest a trial of non-surgical treatment prior to surgical assessment [8, 9] and that the choice to perform surgery should be based on shared decision-making and patient characteristics [7, 10].

In Norway, there was considerable variation (2.1-fold) in decompression rates for LSS between geographical regions from 2012 to 2016 [11]. Also, a US study evaluating 6,265 Medicare beneficiaries diagnosed with LSS showed that patients receiving surgery for LSS were healthier, younger and more likely to be male than those not receiving surgery [12]. However, the overall body of literature describing differences in characteristics of patients who receive LSS surgery and those who do not is limited. Also, only a few newer studies have evaluated the variability and determinants of surgery rates over time, and none of these studies sample a Danish population [3, 4, 13].

Denmark has a long tradition of extensively collecting registry data in the secondary care system, allowing for mapping of variability in LSS surgery rates using the entire Danish population (around 6 million). Therefore, this study aimed to (i) compare patient characteristics, sociodemographic characteristics, geographical region and comorbidities between surgical and non-surgical patients diagnosed with LSS in Danish secondary care from 2002 to 2018 and (ii) investigate regional variation and determinants over time.

## Materials and methods

This study was reported according to the STROBE (Strengthening the Reporting of Observational Studies in Epidemiology) cohort checklist [14].

### Design

Retrospective observational study.

### Data source

The study was based on data from the Danish National Patient Register (DNPR) administered by the Danish Health Authorities [15, 16].

The DNPR registers diagnostic codes based on the International Classification of Diseases (ICD-10 or ICD-11) [17] and surgical procedure codes which build on the Danish version of the Nordic Medico-Statistical Committee (NOMESCO) Nordic Classification of Surgical Procedures (NCSP) system [18]. The DNPR registers health events from all Danish hospitals, including diagnoses and surgical procedures. In 2003 it became mandatory for private hospitals to report health events to the DNPR.

### Study population

The study population included patients with a diagnosis of LSS aged 18 years and older who were registered in the DNPR between 1 and 2002, and 31 December 2018. LSS was defined as (i) spinal stenosis (DM48.0) as the primary diagnostic code or (ii) spondylolisthesis (DM43.1) as the primary diagnostic code in combination with spinal stenosis (DM48.0) as the secondary diagnostic code. The diagnosis DM48.0 is primarily used for LSS, but it is possible that patients with cervical or thoracic stenosis were assigned this code. Therefore, patients assigned a surgical procedure code in the cervical or thoracic spine 270 days after or 90 days before LSS diagnosis were excluded to minimise any misclassification.

Patients were classified as ‘surgical’ if a surgical procedure code was registered in addition to the diagnostic code. Surgery included decompression (ABC36, ABC56, ABC66) with or without supplementary fusion (NAG43, NAG44, NAG46, NAG63, NAG64, NAG66, NAG73, NAG74, NAG76). To be included in the LSS population, patients had to live in Denmark in the year of observation and be alive at the end of the observation year. Patients were excluded if diagnoses indicated scoliosis, deformation, inflammation or infection, fracture, tumours, or cancer related to the spine or spinal disease from a secondary disease during the year they were observed. Details of the data collection are described elsewhere [6].

### Variables

Data on LSS diagnostic codes, surgical procedure codes, date, age, sex, working status, income and geographical region in Denmark (North Denmark Region, Central Denmark Region, Region of Southern Denmark, Region Zealand, and Capital Region of Denmark) were collected. Diagnostic codes for comorbidity (primary or secondary diagnoses) were collected if present within five years before the year of LSS diagnosis, in accordance with the ICD-10 diagnostic categories used in the modified Charlson Comorbidity Index [19, 20]. Comorbidities included myocardial infarction, congestive heart failure, peripheral vascular disease, cerebrovascular disease, dementia, chronic pulmonary disease, connective tissue disease, peptic ulcer disease, mild liver disease, diabetes with and without complications, hemiplegia, moderate or severe renal disease, any tumour, leukaemia, lymphoma, moderate or severe liver disease, metastatic solid tumour and AIDS/HIV. As the data collection process began in 2002, the comorbidities were included for patients registered from 2007 and onwards.

### Statistics

First, the number of patients with a diagnosis of LSS registered in the DNPR between 2002 and 2018 was established. Then, the number of LSS patients who received decompression surgery was calculated as the number of surgical procedures between 2002 and 2018. If a patient was assigned more than one diagnostic LSS code or surgical procedure code within the time period, only the first was recorded. The population was then divided into patients with LSS who received surgery (‘surgical group’), and patients with LSS who did not receive surgery (‘non-surgical group’). Patients in the surgical group entered the data in the year of the surgical event whereas patients in the non-surgical group entered the data in the year of the diagnosis. Multivariable logistic regression was used to estimate relative risks (RR) for surgery. A generalised linear model (GLM) with a binomial distribution and a log function as the link function to relate the variables was used to obtain the RRs directly. The RRs were reported with 95% confidence intervals (CI). The model included indicators of age groups (18–64, 65–74, ≥ 75 [reference group]), sex, region of residence (North Denmark Region [reference group], Central Denmark Region, Region of Southern Denmark, Region Zealand and Capital Region), retirement and income quartile (1st [reference group], 2nd, 3rd and 4th ). Each of the five most common comorbidities from the Charlson Comorbidity Index were identified and included in a separate multivariable logistic regression analysis together with the abovementioned variables. As the comorbidity variables were calculated with a five-year look-back period (the first estimate dates from 2007), the regression including comorbidity variables was restricted to the period 2007–2018. For comparison, the analysis was repeated for 2007–2018, excluding the comorbidity variables. As age, retirement and income are considered to be highly associated, the variance inflation factors (VIFs) were calculated to assess whether this potentially affected the estimates, and sensitivity analyses were performed where appropriate.

To measure variability over time, the RR was calculated for each year by age group, sex, comorbidities, and geographical region. Patients were included in the non-surgical group the year they were diagnosed with LSS, and in the surgical group in the year of surgery. If a patient was assigned more than one diagnostic or surgical code between 2002 and 2018, only the first code was recorded. The annual rates of LSS with and without surgery per 100,000 inhabitants in Denmark on 1 January each year were also reported. Information on the general population is publicly available from Statistics Denmark. The results were presented graphically.

Three periods with different patterns of LSS surgery rates have been previously identified [6]. Surgery rates increased between 2002 and 2010, then decreased between 2011 and 2014, followed by an increase between 2015 and 2018 [6]. We therefore divided the study population into these three periods, i.e. 2002–2010; 2011–2014; 2015–2018. The period 2002–2010 was restricted to 2007–2010 to allow for the inclusion of comorbidity variables. The three periods were then compared using a multivariable logistic regression model to estimate the RR of surgery, including the same variables as in the regression model described above.

## Results

A total of 83,783 unique patients diagnosed with LSS between 2002 and 2018 were identified. Of these, 38,362 (46%) underwent surgery. The majority (n = 34,908) had surgery once, 3,119 had surgery twice, and 335 had surgery three or more times. The mean age of surgical patients was 67 years (SD 11), and 55% were women, which was almost similar to the non-surgical patients (mean age of 66 years (SD 13), 54% women). The majority of LSS patients were aged 65 years and older in both the surgical (62%) and the non-surgical group (59%) (see Table 1). Of the 83,783 included patients, 8,035 were younger than 50 years old.

**Table 1:** Characteristics of patients with LSS by surgical status (2002–2018)

	Total	%	LSS with surgery	%	LSS without surgery	%
n	83,783		38,362		45,421	
**Age**						
18–64	33,173	40	14,507	38	18,666	41
65–74	28,203	34	13,943	36	14,260	31
≥ 75	22,407	27	9,912	26	12,495	28
**Sex**						
Women	45,607	54	21,033	55	24,574	54
Men	38,176	46	17,329	45	20,847	46
**Retirement**						
Retired	59,379	71	27,902	73	31,477	69
Not retired	24,404	29	10,460	27	13,944	31
**Geographical Region**						
Capital Region of Denmark	27,559	33	11,981	31	15,578	34
Central Denmark Region	18,578	22	8,048	21	10,530	23
North Denmark Region	5,335	6	2,672	7	2,663	6
Region Zealand	13,616	16	6,669	17	6,947	15
Region of Southern Denmark	18,695	22	8,992	23	9,703	21
**Comorbidities (2007–2018 only)**						
n	68,449		30,664		37,785	
Diabetes	5,386	8	2,301	8	3,085	8
Chronic obstructive pulmonary disease	4,638	7	1,887	6	2,751	7
Cancer	4,580	7	1,978	7	2,602	7
Peripheral vascular disease	3,425	5	1,480	5	1,945	5
Cerebrovascular disease	3,653	5	1,467	5	2,186	6
**Number of comorbidities mentioned above**						
None	50,857	74	23,116	75	27,741	73
1	14,079	21	6,189	20	7,890	21
≥ 2	3,513	5	1,359	4	2,154	6

Percentages may not total 100 due to rounding.

Patients aged 65 to 74 years were about 12% more likely to receive surgery when compared to those aged 75 years or older (Table 2). Although almost 10% more women than men were diagnosed with LSS (Table 1), women were only 3% more likely to receive surgery when compared to men. We found substantial regional variation in the likelihood of receiving surgery. Patients in the North Denmark Region were more likely to receive surgery than those from other regions. Patients in the Capital Region of Denmark were the least likely to receive surgery (RR 0.84 (CI 0.82; 0.87)), followed by the Central Denmark Region (RR 0.87 (CI 0.84; 0.90)). Retired patients were more likely to receive surgery than those not retired (RR 1.09 (CI 1.06; 1.11)), as were patients in the highest income quartile compared to those in the lowest income quartile (RR 1.11 (CI 1.08; 1.13)).

Patients with the five most common comorbidities (diabetes, chronic obstructive pulmonary disease, cancer, peripheral vascular disease or cerebrovascular disease) were consistently less likely to receive surgery compared to those not inflicted with the specific disease. The minor differences between columns 1 and 2 in Table 2 are due to the different periods rather than from accounting for comorbidities (results available upon request).

**Table 2:** Relative risk for surgery without comorbidity (2002–2018) and in a subpopulation with comorbidity (2007–2018)

	2002–2018		2007–2018 with comorbidities	
	RR	95% CI	RR	95% CI
**Age**				
Age ≥ 75 (reference)	1.00	-	1.00	-
Age 65–74	1.12	1.10;1.14	1.11	1.08; 1.13
Age 18–64	1.01	0.99; 1.04	1.00	0.97; 1.02
**Gender**				
Male	0.97	0.96; 0.99	0.98	0.96; 1.00*
**Region**				
North Region Denmark (reference)	1.00	-	1.00	-
Central Denmark Region	0.87	0.84; 0.90	0.84	0.81; 0.87
Region of Southern Denmark	0.95	0.92; 0.98	0.90	0.87; 0.93
Capital Region of Denmark	0.84	0.82; 0.87	0.81	0.79; 0.84
Region Zealand	0.97	0.94; 1.00	0.96	0.93; 0.99
**Retirement**				
Retired	1.09	1.06; 1.11	1.08	1.06; 1.11
**Income quartiles**				
Income Q1 (reference)	1.00	-	1.00	-
Income Q2	1.00	0.98; 1.02	1.00	0.97; 1.02
Income Q3	1.03	1.01; 1.05	1.04	1.01; 1.06
Income Q4	1.11	1.08; 1.13	1.10	1.07; 1.13
**Comorbidities**				
Diabetes			0.97	0.94; 1.00*
Chronic obstructive pulmonary disease			0.91	0.88; 0.95
Cancer			0.96	0.93; 1.00*
Peripheral vascular disease			0.96	0.93; 1.00*
Cerebrovascular disease			0.90	0.86; 0.93
N	83,772		68,443	

Notes: The analysis that includes comorbidity was performed on data from 2007 to 2018 because comorbidities were registered up to five years prior to index data (Table 2, column 2). A sensitivity analysis without the comorbidity variables was performed on the same data (2007–2018), showing only minor differences on the third decimal (data not shown). 11 patients are excluded due to missing information on income. CI: confidence interval, RR: relative risk, Q: quartile. *CI does not contain 1 but is displayed this way due to rounding

### Variation in the relative risk of surgery over time (2002–2018)

**Fig. 1:**
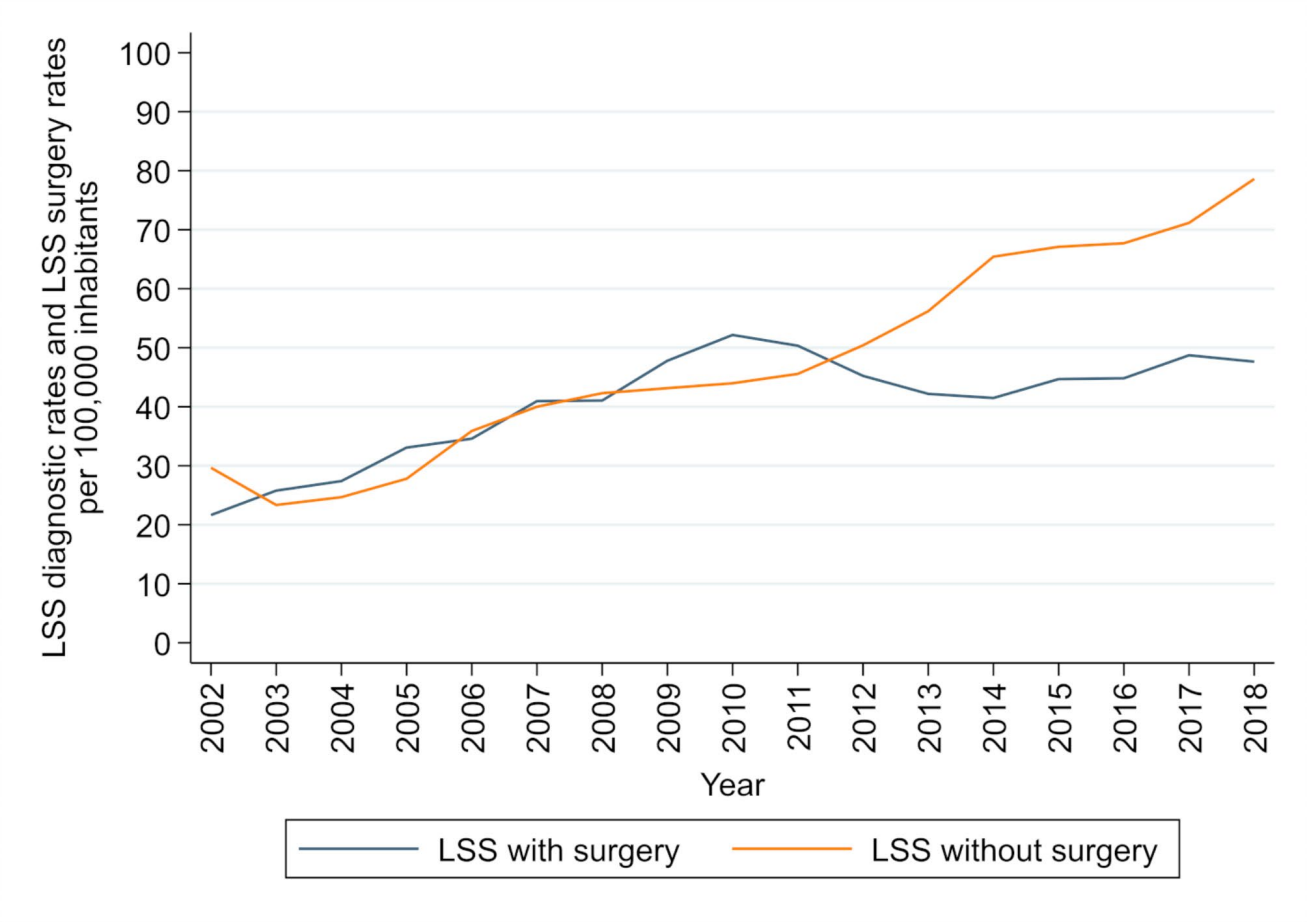
**LSS diagnosis and LSS decompression surgery rates from 2002 to 2018 per 100,000 inhabitants**

Overall, the rate of LSS diagnosis per 100,000 inhabitants increased steadily over time, whereas the rate of LSS surgery started to decrease around 2010, followed by an increase around 2014 (Fig. 1).

**Fig. 2:**
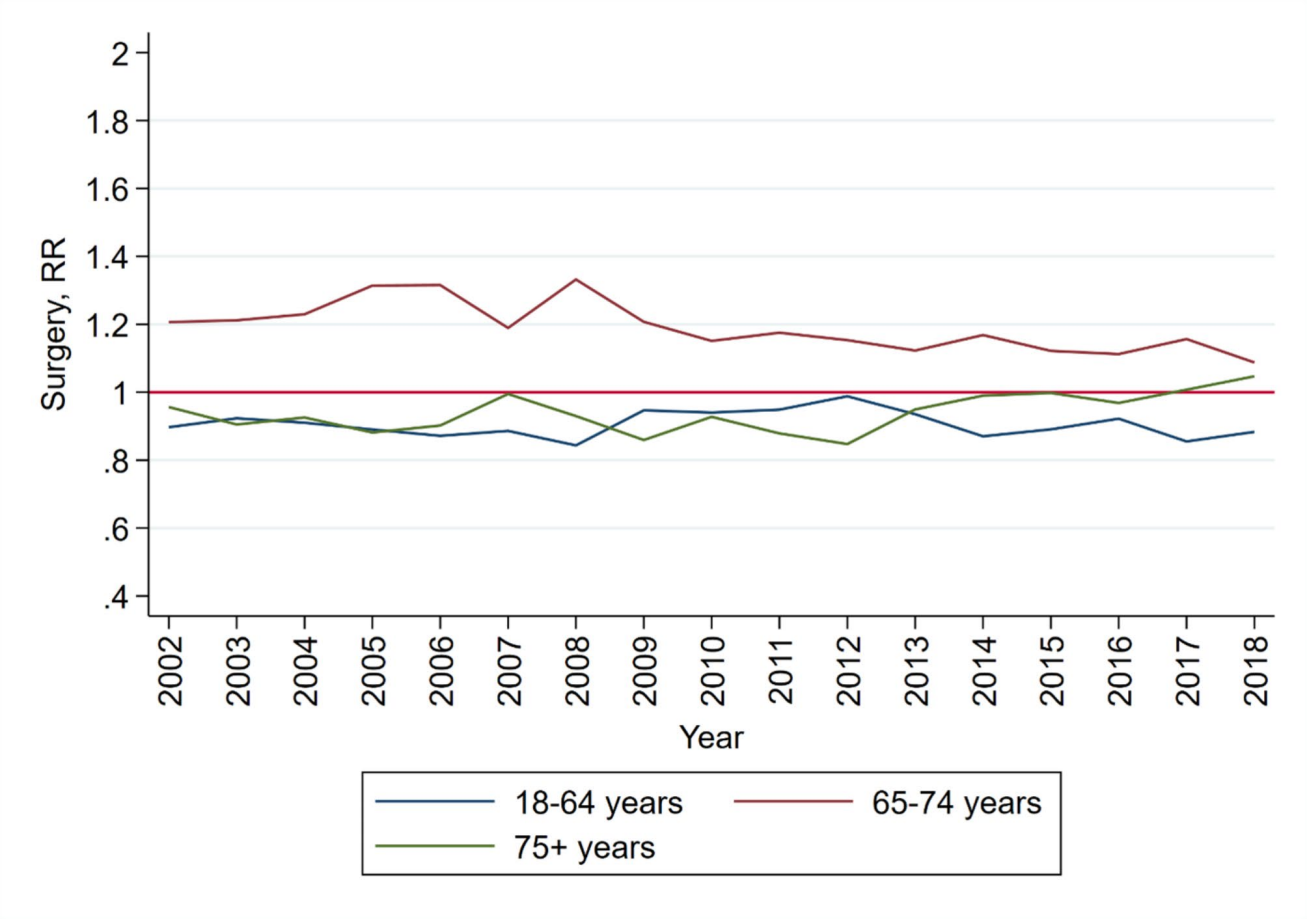
**Relative risk of LSS patients having surgery from 2002 to 2018, stratified by age groups**. The graph shows the unadjusted relative risks for each age group.

Over time, the likelihood of patients aged 65–74 years undergoing surgery decreased slightly, and a minor increase was observed for patients aged 75 years or older (Fig. 2).

There were no differences over time between men and women in those who received surgery and those who did not. The pattern showing that patients with one of the five most common comorbidities were less likely to have surgery than patients without one of these diagnoses remained relatively stable over time.

**Fig. 3:**
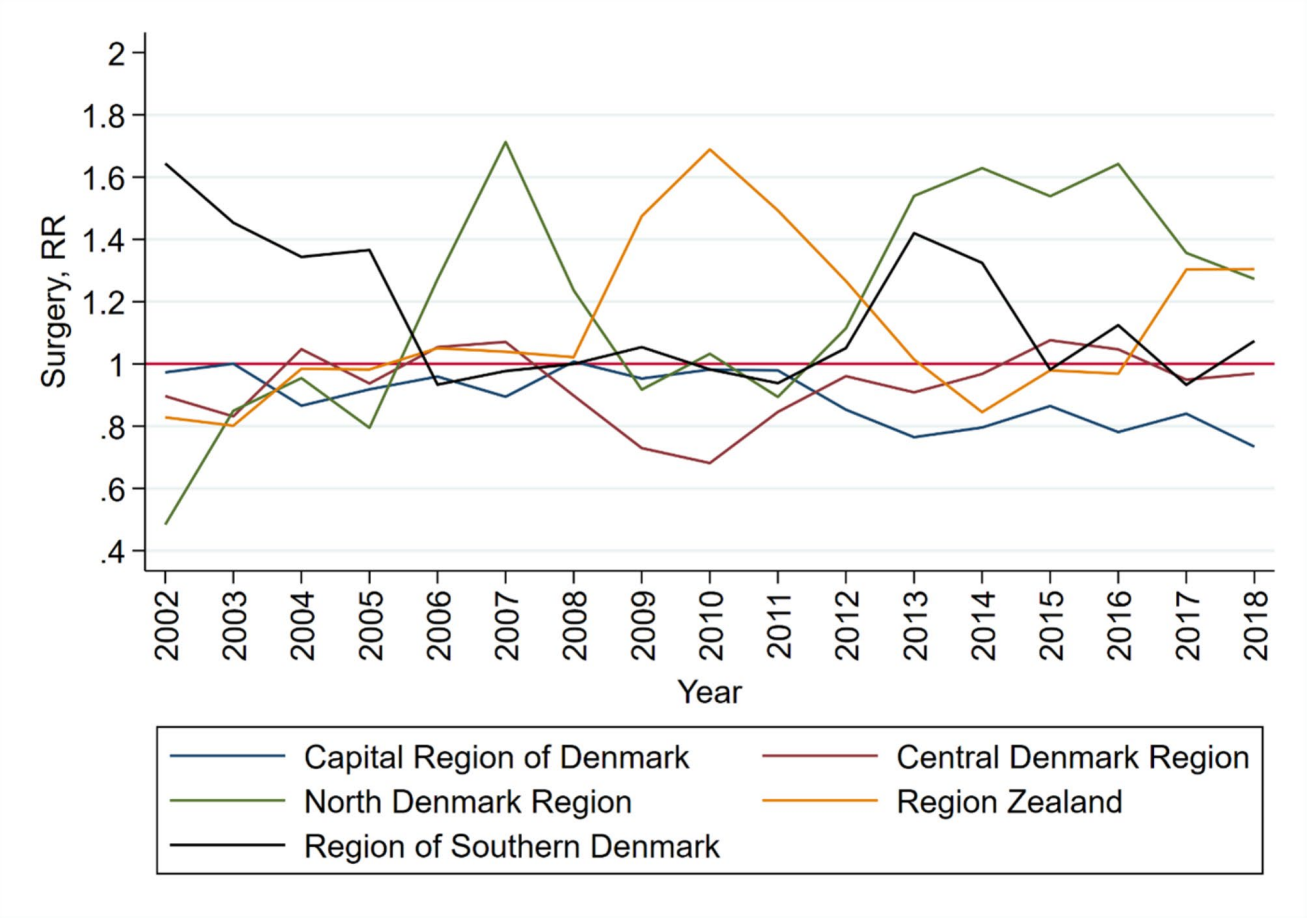
**Relative risk of LSS patients having surgery from 2002 to 2018, stratified by geographical region**. The graph shows the unadjusted relative risks for each region.

Considerable differences and fluctuations in the relative risk of surgery were observed within and between the geographical regions (Fig. 3). In the Region of Southern Denmark, patients were more likely to have surgery between 2002 and 2005 and again in 2013–2014, with a 60% and 40% increase, respectively, compared to patients from other regions in the same periods. In 2007 and again in 2013–2016, patients in the North Denmark Region were 60–70% more likely to have surgery than patients from other regions. In 2009–2011, patients in Region Zealand had a 50–70% higher likelihood of surgery than patients in other regions. The relative risk in the Capital Region of Denmark and the Central Denmark Region showed a more stable pattern over time.

The results describing variation in the relative risk of surgery over time have been presented visually in Additional file 1 as detailed graphs of the variation in the surgical and non-surgical populations per age group, sex, comorbidities and geographical region as index 100 and relative risk. The surgery rates per 100,000 inhabitants stratified by region are also presented in Additional file 1, showing large variations in surgery rates per 100,000 inhabitants with the most extensive difference ranging from 42 to 102 per 100,000 in 2010.

As shown in Fig. 1, surgery rates increased until 2010, then decreased between 2011 and 2014, followed by an increase between 2015 and 2018. Based on this pattern, the data were divided into three corresponding time intervals (2007–2010, 2011–2014 and 2015–2018). Patients aged 65–74 years were more likely to receive surgery during all three periods (Table 3). However, age group differences diminished over time as older patients (≥ 75) increasingly often underwent surgery. The likelihood of patients in the Capital Region of Denmark (relative to the North Denmark Region) having surgery performed declined from an 8% lower likelihood in the first period (2007–2010) to 28% lower in the latest period (2015–2018), while surgery rates in the Central Denmark Region remained constant at about a 16% lower likelihood. Retired patients were 15% more likely to receive surgery during the first period, whereas in the last period, we found no significant difference compared to non-retirees. Those with a higher income were more likely to receive surgery, although this difference diminished over time. Finally, patients with comorbidities were less likely to receive surgery. However, only patients with chronic obstructive pulmonary disease (COPD) were at statistically significant lower relative risk during all three periods.

**Table 3:** Relative risk for surgery (2007–2018) by three time periods

	2007–2010		2011–2014		2015–2018	
	RR	95% CI	RR	95% CI	RR	95% CI
**Age**						
Age ≥ 75 (reference)	1.00	-	1.00	-	1.00	-
Age 65–74	1.14	1.10; 1.18	1.13	1.09; 1.17	1.06	1.02; 1.09
Age 18–64	1.04	1.00; 1.09	1.01	0.96; 1.06	0.92	0.89; 0.97
**Gender**						
Male	0.95	0.93; 0.98	0.97	0.95; 1.00	1.02	0.99; 1.05
**Region**						
North Region Denmark (reference)	1.00	-	1.00	-	1.00	-
Central Denmark Region	0.84	0.79; 0.90	0.84	0.80; 0.90	0.83	0.78; 0.87
Region of Southern Denmark	0.94	0.89; 1.00	0.96	0.90; 1.01	0.83	0.79; 0.88
Capital Region of Denmark	0.92	0.87; 0.98	0.81	0.76; 0.85	0.72	0.68; 0.76
Region Zealand	1.05	0.99; 1.12	0.96	0.90; 1.02	0.89	0.85; 0.94
**Retirement**						
Retired	1.15	1.10; 1.20	1.05	1.00; 1.10	1.04	0.99; 1.09
**Income quartiles**						
Income Q1 (reference)	1.00	-	1.00	-	1.00	-
Income Q2	1.02	0.98; 1.06	0.98	0.94; 1.02	1.00	0.96; 1.04
Income Q3	1.06	1.02; 1.11	1.03	0.98; 1.07	1.01	0.97; 1.06
Income Q4	1.13	1.08; 1.17	1.11	1.06; 1.16	1.05	1.00; 1.10*
**Comorbidities**						
Diabetes	0.95	0.90; 1.0	0.98	0.92; 1.03	0.97	0.92; 1.03
*Chronic obstructive pulmonary disease*	0.92	0.87; 0.98	0.92	0.87; 0.98	0.90	0.84; 0.96
Cancer	0.95	0.90; 1.01	0.95	0.89; 1.01	0.99	0.94; 1.05
*Peripheral vascular disease*	1.01	0.95; 1.07	0.94	0.88; 1.01	0.94	0.87; 1.01
*Cerebrovascular disease*	0.88	0.82; 0.94	0.88	0.82; 0.94	0.93	0.87; 1.00
N surgery	10,000		10,021		10,643	
N total	19,307		22,197		26,939	

Notes: Column 1 includes the population from 2007–2010, column 2 from 2011–2014 and column 3 from 2015–2018. The data are restricted to 2007 and onwards to enable the inclusion of comorbidities. The entire period (2007–2018) is shown in Table 2for reference. Six patients were excluded due to missing information on income. CI: confidence interval, RR: relative risk, Q: quartile. *CI does not contain 1 but is displayed this way due to rounding

## Discussion

Over a 17-year period, 46% of Danish patients diagnosed with LSS eventually underwent surgery. Patients were more likely to have surgery if they were aged 65–74 years, retired, did not have comorbidities and were in a high-income group. We found considerable geographical variation, varying from an approximately 50% decreased likelihood to a 70% increased likelihood depending on geographical region (Fig. 3). The most extensive variation in surgery rates per 100,000 inhabitants was in 2010, ranging from 42 to 102 per 100,000.

Many factors impact surgery rates. For example, limited surgical capacity can result in reduced surgery rates due to longer waiting lists for patients, which in turn may influence prioritisation for surgery, leading to delays or cancellations for less severe cases resulting in longer waiting lists and lower surgery rates. Other factors such as population demand, patient preferences, health policy and contextual factors also shape regional surgery rates.

Historically, surgery has been performed less often in the oldest patient groups, probably because complication rates and post-surgery mortality rates increase with age [21]. However, we observed an increase in surgery rates for our oldest age group over time. This tendency could be due to fewer consequences of age and comorbidities, a development in surgical techniques or an increasing demand among the oldest patients. Another probable contributing factor is a general increased awareness of LSS, as we have previously shown that the growth rate of LSS diagnosis and surgery among the oldest age groups exceeded the growth rate in the general Danish population [6].

Although comorbidity is closely related to increasing age, our results showed that a diagnosis of either diabetes, chronic obstructive pulmonary disease, cancer, peripheral vascular disease or cerebrovascular disease within five years before surgery was independently associated with a decreased likelihood of receiving LSS surgery. This finding is in line with a study by Chen et al. [12], who found that LSS patients undergoing surgery were healthier than non-surgical LSS patients. Patients in the highest income quartile were 13% more likely to receive surgery between 2007 and 2010 compared to the lowest income quartile, but this difference was reduced to less than 5% between 2015 and 2018. The same pattern was observed for patients on retirement, who were 14% more likely to undergo surgery between 2007 and 2010, whereas between 2015 and 2018, the difference was less than 4% and no longer statistically significant. Age, retirement and income are highly associated because increasing age leads to retirement and lower income. A trend towards more elderly receiving surgery could therefore lead to an underestimation of the effect of low income. However, after applying the appropriate model check, our results remained robust.

We found substantial geographical variations in the relative risk of surgery, both within the same region and between the five Danish regions. LSS patients in the North Denmark Region and Region Zealand were most likely to undergo surgery, and these regions also have the largest proportion of inhabitants above 65 years [22]. Although some of this variation can be explained by differences in demography between geographical regions, the differences do not explain the considerable variation over time and certainly not the variation within a specific region. Geographic variations in surgery rates have been reported for other countries, and it has been suggested that variations in surgical indications, organisation and capacity can influence these time trends [1, 5, 23].

It is not reassuring to identify such wide variations across all regions in a small country with only 5.8 million inhabitants and a healthcare system that is predominantly publicly financed. However, each of the five regions owns and administers the regional hospitals, and the regions are not obligated to coordinate health services between them. Some regions are organised with one large, specialised spine hospital with medical and surgical departments, whereas in other regions, spinal pain patients are managed by rheumatology, orthopaedic, neurological and neurosurgical departments based in different hospitals. This difference in organisational structure is likely one cause of variation across regions. Also, it is possible that differences in waiting lists may have influenced the variation. However, data on waiting lists were not available and calculating the time from diagnosis to surgery as a proxy for waiting lists was not considered useful because the time from diagnosis to surgery vary dependent on many factors such as a period of conservative management, postponement of surgery while observing potential improvement in symptoms, waiting for treatment of other conditions, or patients requesting time to consider whether or not to have surgery.

Systematic variation in the utilisation of health care services are well established [24, 25], and when such variations are due to changes in a patient’s health status, treatment preferences, or need for medical care, they are considered warranted. However, if the variation is due to factors unrelated to a patient’s medical needs, it is considered unwarranted. Wennberg et al. [26] proposed the theory of unwarranted variation in healthcare utilisation, which describes three types of unwarranted variation: effective care, preference-sensitive care and supply-sensitive care. *Effective care* refers to clinicians’ underutilisation of evidence-based interventions, while *preference-sensitive* care is influenced by patient preferences and values. *Supply-sensitive care* is influenced by the availability of healthcare resources. Addressing unwarranted variation can lead to more efficient and patient-centred care.

There are both strengths and limitations to registry-based studies. The registries contain data from all private and public hospitals in Denmark. Therefore, it is possible to determine the rates of diagnostic and surgical procedures across different geographical regions with good precision. The registries include at least 95% of LSS surgeries in Denmark [16], and the completeness of DNPR has previously been validated [27]. However, there could be differences in the use of the diagnostic code for LSS, depending on the type of secondary care centre the patient attended for examination and treatment. For example, surgical and rheumatology departments may use diagnostic codes differently, despite similar patient presentations, resulting in misclassification [27]. The diagnostic code of LSS used in this study (ICD-10 DM48.0) is used for the lumbar region but could, in some cases, be used for cervical or thoracic stenosis. However, the likelihood of this affecting the study is minor when excluding surgical procedures for cervical and thoracic regions, as described in the methods section. Although we excluded surgical procedure codes for cervical and thoracic regions, the cohort could contain patients misclassified with stenosis in other anatomical regions. This could introduce an uncertainty in the exact estimates, but as the proportion of misclassifications is unlikely to vary over time, the results can provide reliable estimates of time trends.

## Conclusion

Over a 17-year period, 46% of patients diagnosed with LSS in Danish secondary care underwent LSS surgery. There were considerable variations in the relative risk of surgery between and within geographical regions. Patients aged 65 to 74 years were more likely to receive surgery than other age groups, and LSS surgical patients were healthier, more often retirees, and had higher incomes than those who did not undergo surgery.

## Electronic supplementary material

Below is the link to the electronic supplementary material.

Additional file 1: Development in diagnosis and surgery rates of lumbar spinal stenosis stratified by age groups, sex, geographical region and comorbidity displayed graphically

## Data Availability

The data that support the findings of this study are available from Statistics Denmark’s Research Service, but restrictions apply to the availability of these data, which were used under licence for the current study, and thus are not publicly available. Data are, however, available from the authors upon reasonable request and with permission of Statistics Denmark’s Research Service.

## List of abbreviations

CEVD: Cerebrovascular disease
CI: Confidence interval
COPD: Chronic obstructive pulmonary disease
DNPR: Danish National Patient Register
ICD: International classification of diseases
LSS: Lumbar spinal stenosis
NCSP: Nordic classification of surgical procedures
NOMESCO: Nordic Medico-Statistical Committee
PVD: Peripheral vascular disease
Q: Quartile
RR: Relative risk
SD: Standard deviation
