# Supplementary material for: Surgical trends and regional variation in Danish patients diagnosed with lumbar spinal stenosis between 2002 and 2018: a retrospective registry-based study of 83,783 patients
Source: BMC Health Serv Res. 2023 Jun 20;23:665. doi: 10.1186/s12913-023-09638-7 (PMC10283336; doi:10.1186/s12913-023-09638-7)

Additional file 1. Development in diagnosis and surgery rates of lumbar spinal stenosis stratified by age groups, sex, geographical region and comorbidity

Development in rates of LSS patients with and without surgery from 2002 to 2018 stratified by age groups

Age groups

Rates of surgery and diagnoses (index 100)	Relative risk of surgery

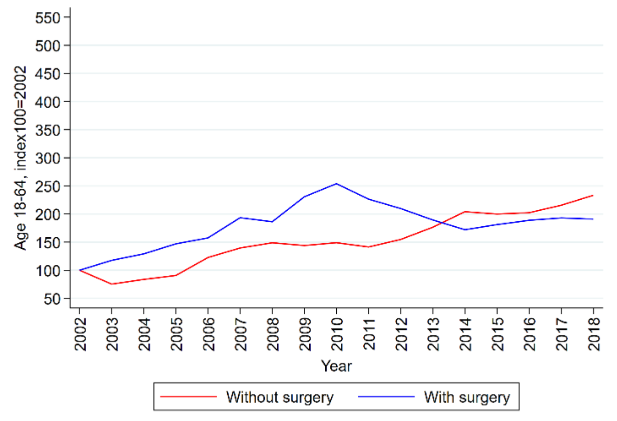

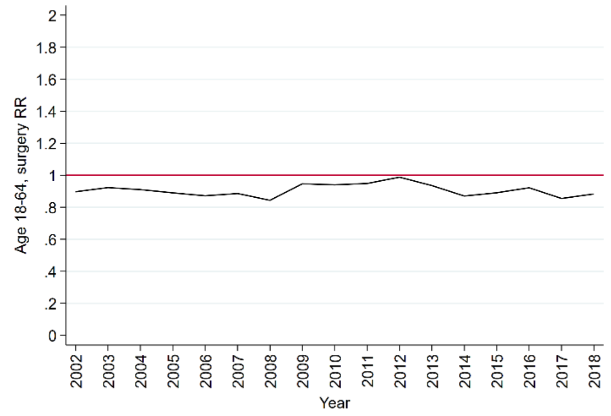

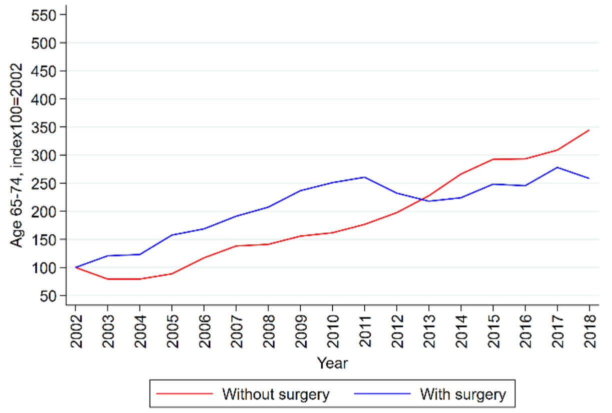

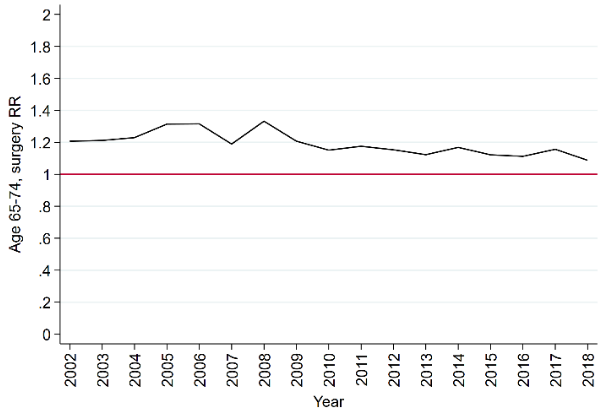

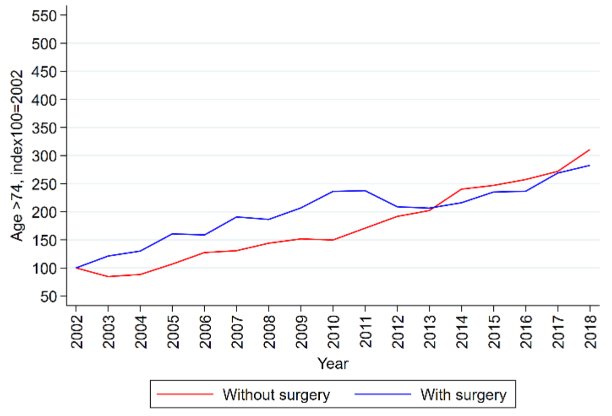

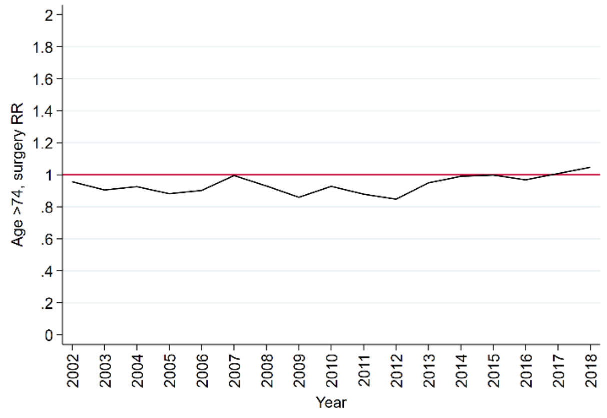

Development in rates of LSS patients with and without surgery from 2002 to 2018 stratified by sex

<table>
<tr><th colspan="2">Sex</th></tr>
<tr><th>Rates of surgery and diagnoses (index 100)</th><th>Relative risk of surgery</th></tr>
<tr><td>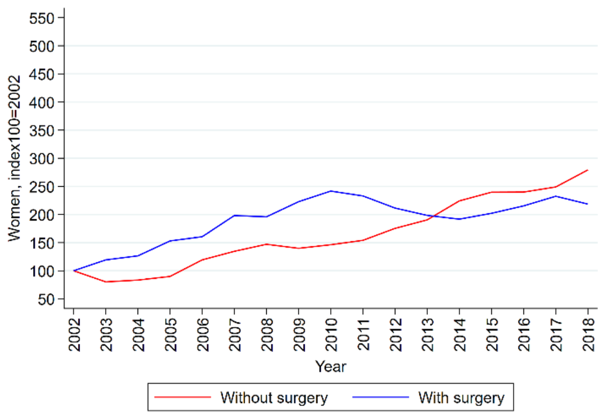
</td><td>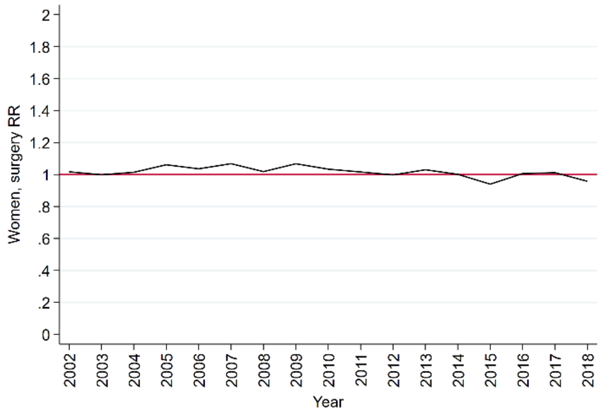
</td></tr>
<tr><td>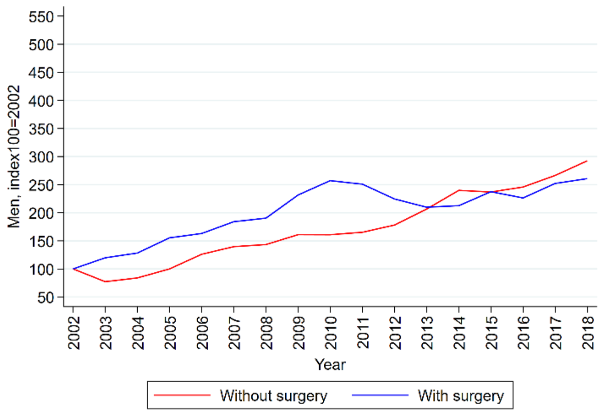
</td><td>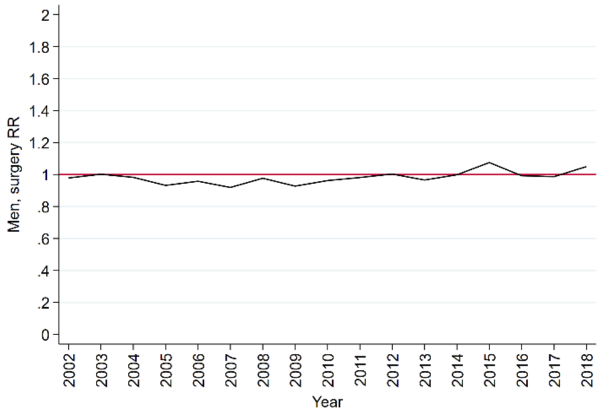
</td></tr>
</table>

Development in rates of LSS patients with and without surgery from 2002 to 2018 stratified by geographical region

Region	
Rates of surgery and diagnoses (index 100)	Relative risk of surgery

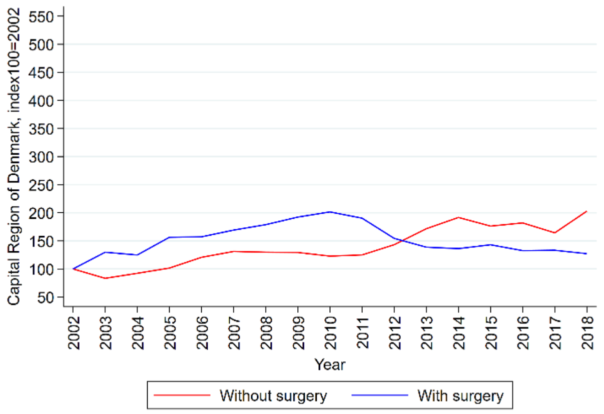

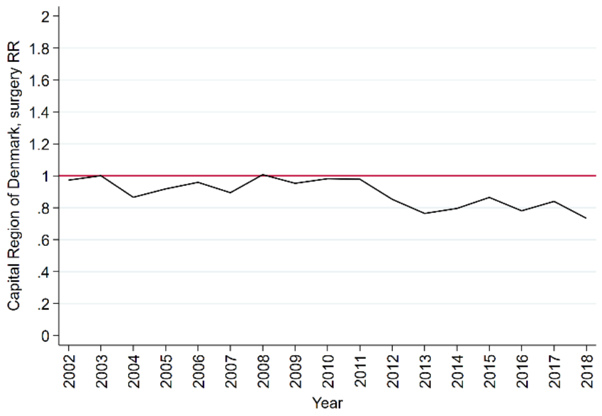

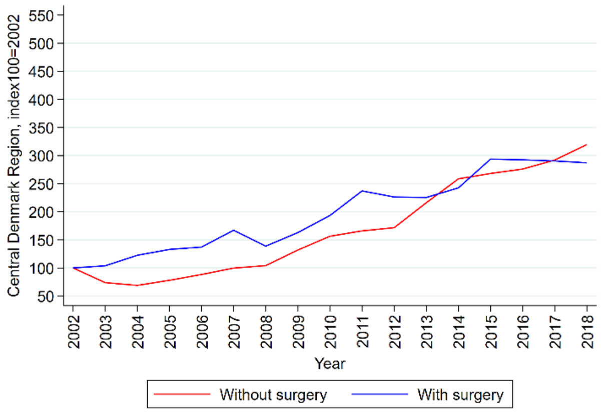

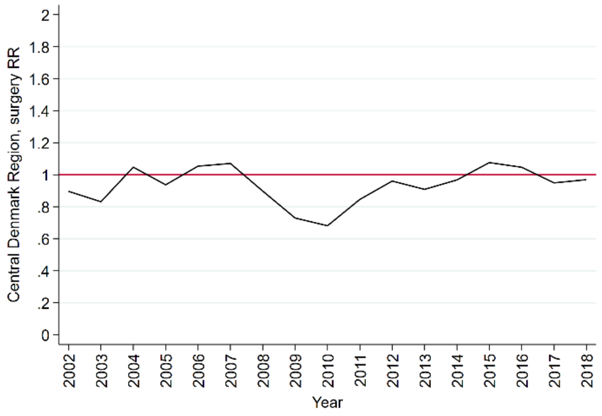

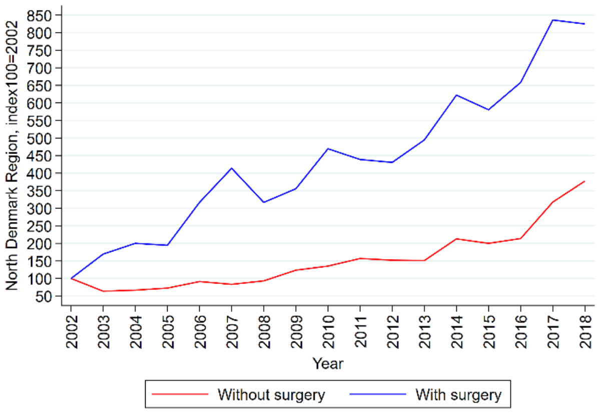

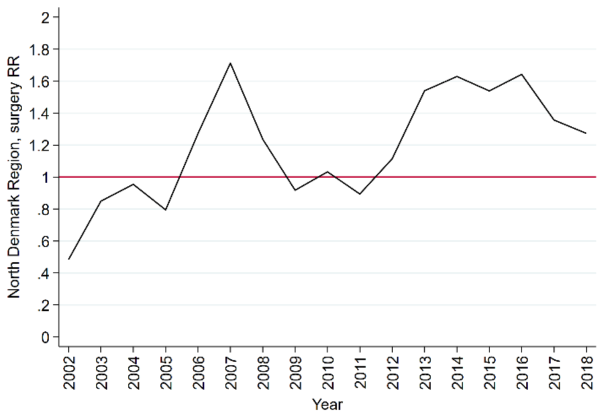

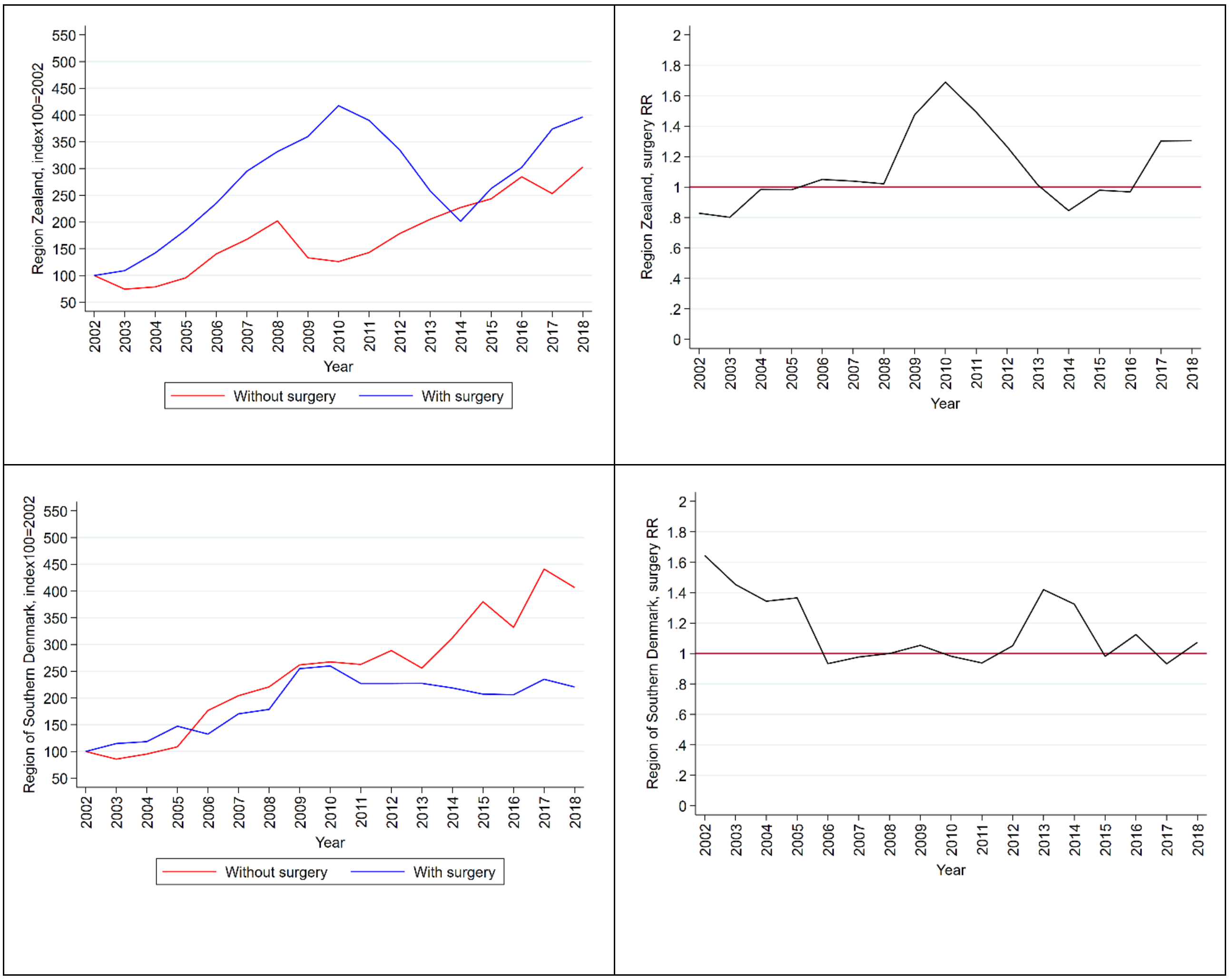

Region Zealand, index100=2002
Year
Without surgery
With surgery
Region Zealand, surgery RR
Year
Region of Southern Denmark, index100=2002
Year
Without surgery
With surgery
Region of Southern Denmark, surgery RR
Year

Development in rates of LSS patients with and without surgery from 2002 to 2018 stratified by comorbidity

Comorbidity

Rates of surgery and diagnoses (index 100)	Relative risk of surgery

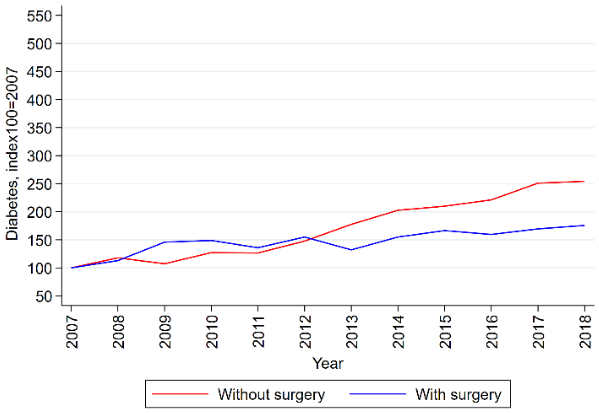

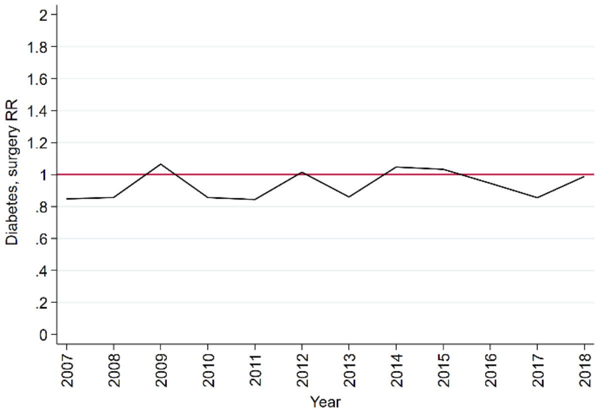

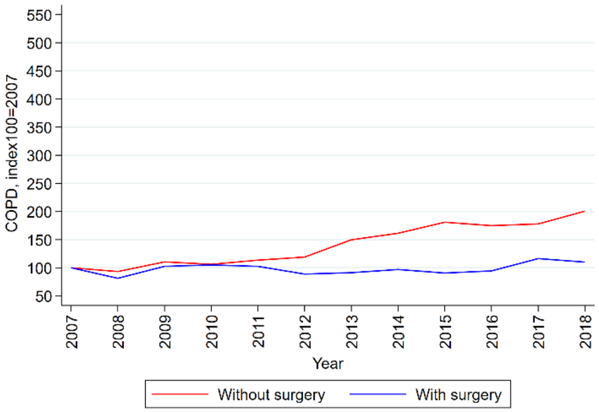

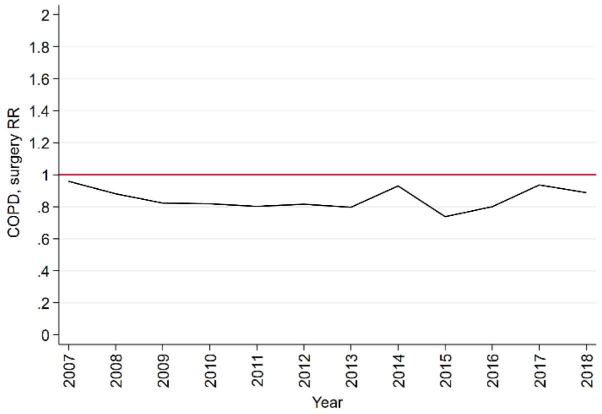

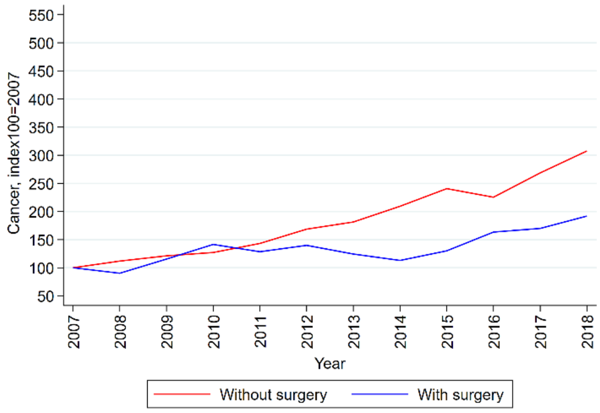

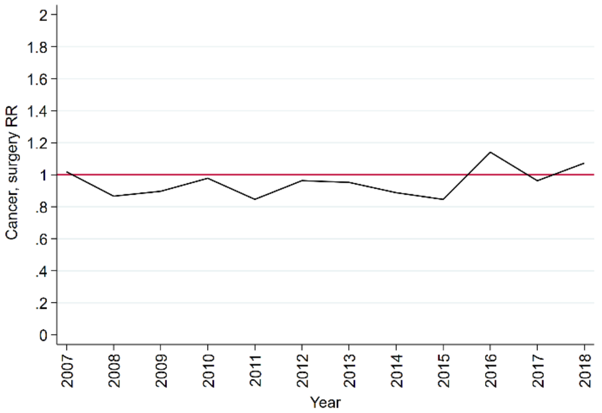

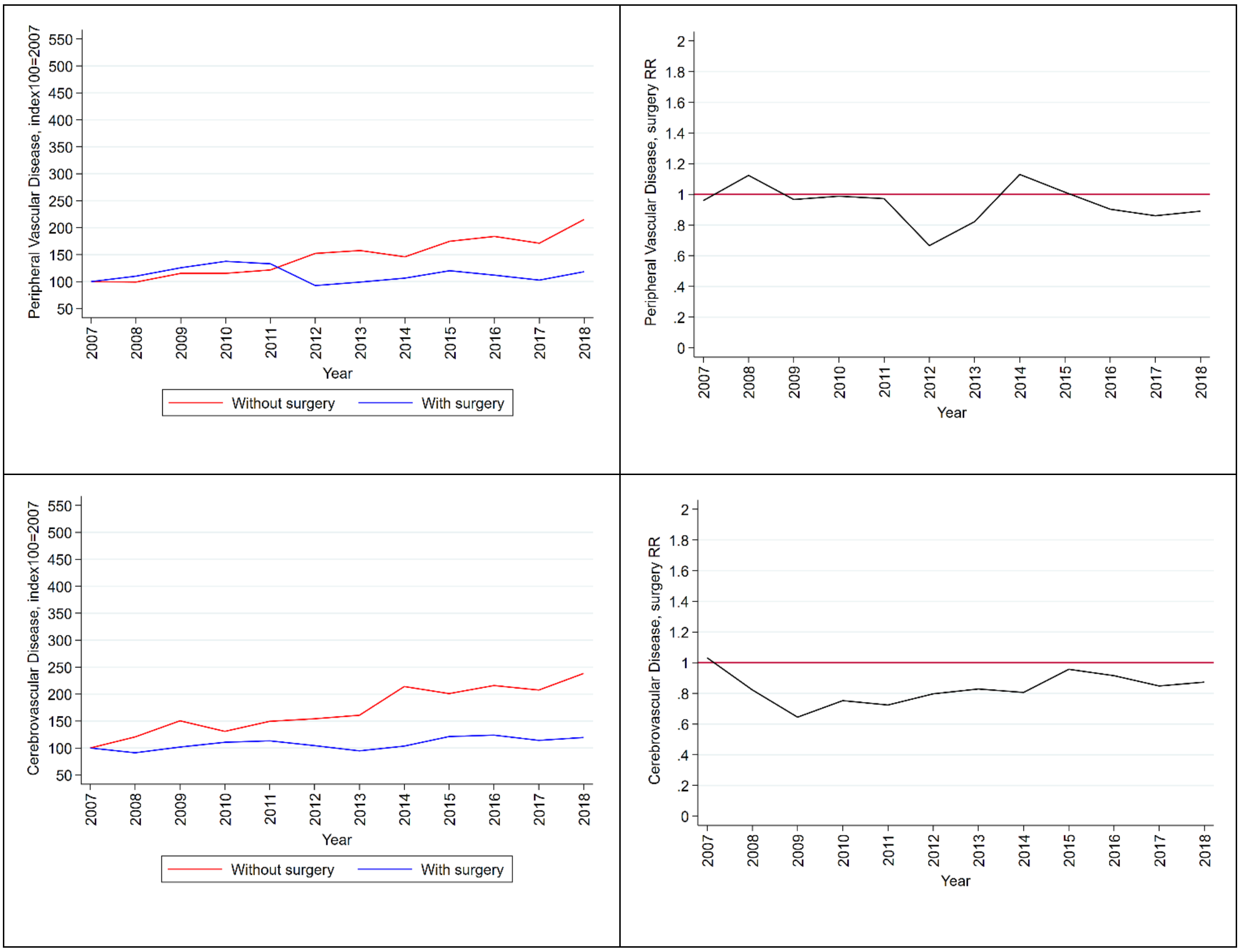
Peripheral Vascular Disease, index100=2007
Year
Without surgery
With surgery
Peripheral Vascular Disease, surgery RR
Year
Cerebrovascular Disease, index100=2007
Year
Without surgery
With surgery
Cerebrovascular Disease, surgery RR
Year

Development in number of LSS surgeries per 100,000 from 2002 to 2018 stratified by geographical region

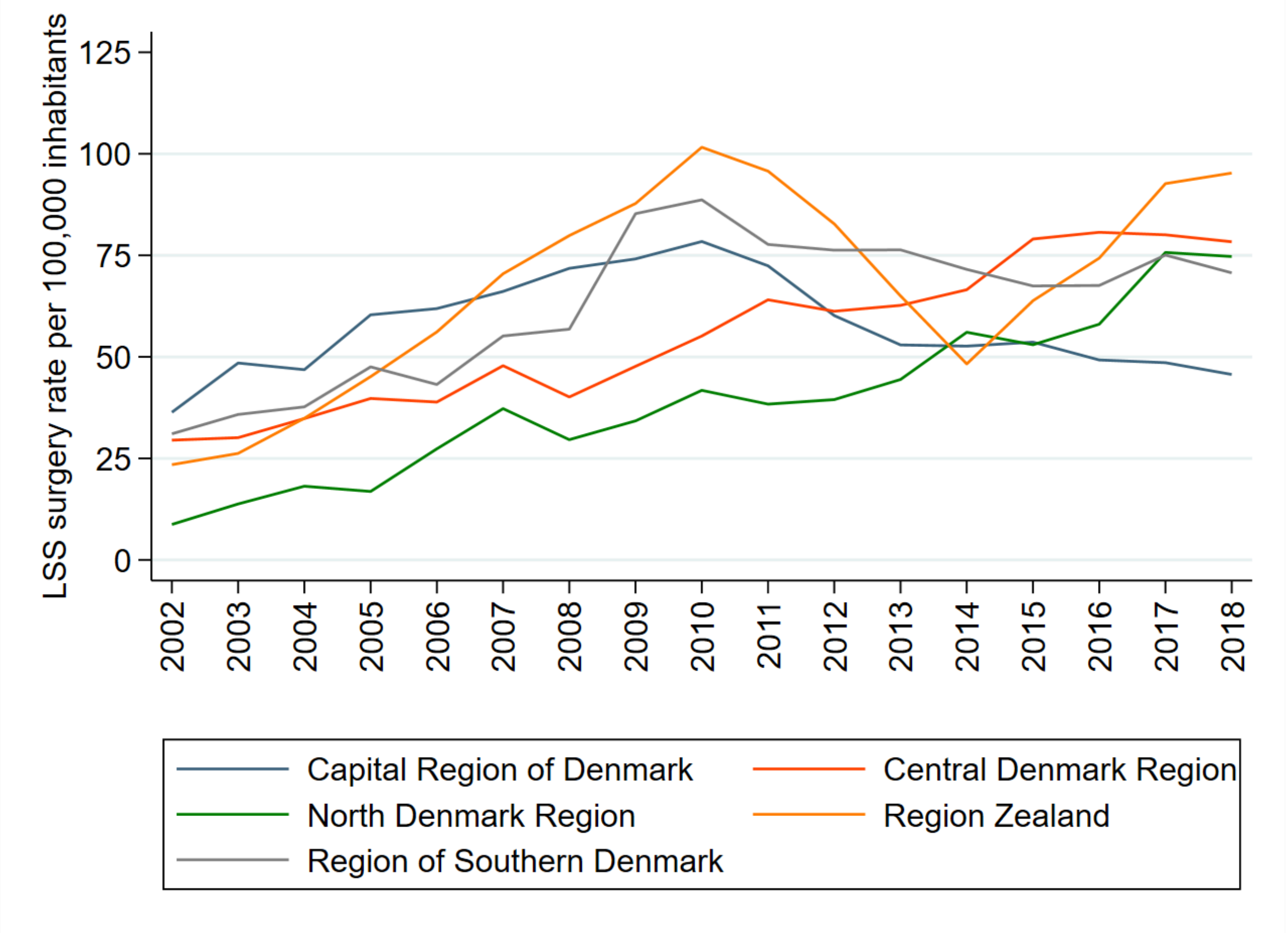

Supplement: Supplementary file 1 — Additional file 1: Development in diagnosis and surgery rates of lumbar spinal stenosis stratified by age groups, sex, geographical region and comorbidity displayed graphically [file 12913_2023_9638_MOESM1_ESM.pdf]
